# Abnormal Dynamic Functional Connectivity Associated With Subcortical Networks in Parkinson’s Disease: A Temporal Variability Perspective

**DOI:** 10.3389/fnins.2019.00080

**Published:** 2019-02-19

**Authors:** Hong Zhu, Juan Huang, Lifu Deng, Naying He, Lin Cheng, Pin Shu, Fuhua Yan, Shanbao Tong, Junfeng Sun, Huawei Ling

**Affiliations:** ^1^Shanghai Med-X Engineering Research Center, School of Biomedical Engineering, Shanghai Jiao Tong University, Shanghai, China; ^2^Department of Radiology, Ruijin Hospital, Shanghai Jiao Tong University School of Medicine, Shanghai, China; ^3^School of Biomedical Engineering, Shanghai Jiao Tong University, Shanghai, China

**Keywords:** Parkinson’s disease, dynamic functional connectivity, resting-state fMRI, subcortical networks, temporal variability

## Abstract

Parkinson’s disease (PD) is a neurodegenerative disease characterized by dysfunction in distributed functional brain networks. Previous studies have reported abnormal changes in static functional connectivity using resting-state functional magnetic resonance imaging (fMRI). However, the dynamic characteristics of brain networks in PD is still poorly understood. This study aimed to quantify the characteristics of dynamic functional connectivity in PD patients at nodal, intra- and inter-subnetwork levels. Resting-state fMRI data of a total of 42 PD patients and 40 normal controls (NCs) were investigated from the perspective of the temporal variability on the connectivity profiles across sliding windows. The results revealed that PD patients had greater nodal variability in precentral and postcentral area (in sensorimotor network, SMN), middle occipital gyrus (in visual network), putamen (in subcortical network) and cerebellum, compared with NCs. Furthermore, at the subnetwork level, PD patients had greater intra-network variability for the subcortical network, salience network and visual network, and distributed changes of inter-network variability across several subnetwork pairs. Specifically, the temporal variability within and between subcortical network and other cortical subnetworks involving SMN, visual, ventral and dorsal attention networks as well as cerebellum was positively associated with the severity of clinical symptoms in PD patients. Additionally, the increased inter-network variability of cerebellum-auditory pair was also correlated with clinical severity of symptoms in PD patients. These observations indicate that temporal variability can detect the distributed abnormalities of dynamic functional network of PD patients at nodal, intra- and inter-subnetwork scales, and may provide new insights into understanding PD.

## Introduction

Parkinson’s disease (PD) is a progressive neurodegenerative disease that affects millions of people around the world. The prominent symptoms in PD are the motor deficits including tremor, rigidity, and bradykinesia, which are mainly due to the loss of dopaminergic neurons in the substantia nigra (Kalia and Lang, 2015; Ma et al., 2016). Previous studies had proposed that the dysfunction of corticostriatal network pathways and neural circuits is related to the impairments in PD (Hacker et al., 2012; Agosta et al., 2013; Luo et al., 2014; Amboni et al., 2015; Alia et al., 2016). Despite being extensively investigated, mechanisms underlying disturbances in PD are still poorly understood.

The past decade has seen remarkable growth of network neuroscience and neuroimaging techniques and their applications to abnormal brain connectivity in psychiatric and neurological disorders. Several previous studies highlighted that PD could be considered as a disease related to the disruptions in several networks using diffusion tensor imaging (DTI) (Melzer et al., 2013; Lopes et al., 2017), resting-state functional magnetic resonance imaging (fMRI) (Luo et al., 2014), task fMRI (Shine et al., 2013b) and other imaging techniques (Brooks and Pavese, 2011). Functional network analysis based on resting-state fMRI data, accounting for the intrinsic blood oxygen level-dependent (BOLD) fluctuations, offers an effective tool for characterizing interactions between brain regions and quantifying abnormal organization of brain network in disorders (Bullmore and Sporns, 2009; Smith et al., 2009, 2011). Previous studies on large-scale network of PD patients by graph theoretic analysis revealed disruptions in the topological properties of brain networks and these network measures have been demonstrated to serve as potential biomarkers of PD for clinical diagnosis (Amboni et al., 2015). Furthermore, altered modular organization of functional brain networks in PD patients has also been reported (Ma et al., 2016; Peraza et al., 2017), implying an abnormal functional integration of PD.

However, the majority of earlier studies have adopted a “static” point of view, whereas functional connectivity (FC) between regions are actually associated with dynamic brain activity over time (Hutchison et al., 2013; An et al., 2017). Dynamic functional connectivity (dFC) analysis has been directed to assess relevant FC fluctuations and examine how functional organization evolves over time. It sheds new insights on the dynamic spatiotemporal organization of resting brain activity and captures FC alterations induced by disease pathologies (Preti et al., 2016; Khambhati et al., 2017). For example, previous studies have found that Schizophrenia (SZ) patients displayed within-network disruptions of the DMN (Du et al., 2016) as well as weaker across-network connectivity between DMN and other resting-state networks (RSNs) (Rashid et al., 2014; Su et al., 2016). In addition, the dynamic connectivity patterns have served as features to gain better identification output in the classification of MCI subjects (Wee et al., 2016) and of autism patients (Price et al., 2014) than the standard static approaches. These results suggest that the abnormal network characteristics of PD may emerge from dynamic functional connections that cannot be completely captured by static approaches and may help deepen our understanding of this disease.

Despite these applications, alterations in the dynamic properties of PD individuals still remain largely unknown. Kim et al. (2017) first investigated the temporal states of dFC and variability of network topology in PD patients using k-means clustering, and found that PD patients showed a decrease in the sparsely connected State I, paralleled by an increase in the stronger interconnected State II, suggesting the altered functional segregation and abnormal global integration in brain networks. Sourty et al. (2016) employed Product Hidden Markov Models (PHMM) to assess the connectivity state changes between a set of RSNs in dementia with Lewy bodies, a disease sharing similar features to PD, and figured out networks (i.e., occipito-parieto-frontal network, the medial occipital network and the right fronto-parietal network) related to impairment of cognitive function in patients. However, these studies mainly focused on the connectivity state changes of the whole brain, ignoring the dynamic connectivity profile of particular brain regions. A recent work (Zhang et al., 2016) investigated the temporal properties of dFC by defining the temporal variability of FCs associated with a specific brain region, and the temporal variability reveled a strong correlation with BOLD/EEG activity. This approach allows localization of regions showing significant variability between groups, thus helping to reveal the abnormality of regional dynamics of functional brain networks in various brain diseases. Another work (Jie et al., 2018) integrated both temporal and spatial variabilities of dynamic functional networks for automatic diagnosis of Alzheimer’s Disease and boosted the diagnosis performance, demonstrating that the spatio-temporal interaction patterns can provide important information on the underlying nature of neurodegenerative disease. Actually, PD is a disease associated with a disruption across diffuse areas of brain and interactions of multiscale organization. However, the temporal properties of subnetworks (e.g., the intra- and inter-network variability) have not been investigated yet. Temporal variability may help elucidate the aberrant changes underlying PD patients from the perspective of regional dynamics of functional brain networks. A systematic examination of dFC patterns and temporal variability in aspects of nodal, intra- and inter- subnetworks may further deepen our understanding of PD.

In this study, we performed dFC analysis built on non-overlapping networks to investigate the aberrant dFC patterns in PD patients. With a focus on temporal variability of FC profiles, we systematically examined the dFC changes at nodal, intra- and inter- subnetwork levels. Our major goal was to demonstrate whether: (1) the temporal variability could characterize the underlying alterations in the PD cohort and/or (2) the changes in temporal variability could account for some clinical symptoms of PD.

## Materials and Methods

### Participants and Assessment

Forty-four PD subjects were recruited from Ruijin Hospital affiliated to Shanghai Jiao Tong University. The inclusion criteria included: (i) a diagnosis of PD without any record of other neurological or psychiatric disorders; (ii) age ≥ 45 years; (iii) Mini Mental State Examination [MMSE (Folstein et al., 1975)] ≥ 24; (iii) no depressive symptoms [evaluated by Beck Depression Inventory (BDI) (Beck et al., 1961)]. Clinical evaluation was assessed with Hoehn and Yahr (H-Y) score (Hoehn and Yahr, 1998) for the disease stage and the Unified Parkinson’s Disease Rating Scale-Part III (UPDRS-III) (Chai et al., 2017) for severity of motor symptoms. Forty-three normal controls (NCs) were also included for the study. Three normal participants with head motion >2 mm of translation or >2 degree of rotation and two PD patients with poor co-registration in cerebellum were excluded in the following analysis (see section “Data Preprocessing” for details), leaving 42 patients and 40 normal controls for analysis. The relevant demographic and clinical information are summarized in Table 1. This study was carried out in accordance with the recommendations of the “ethics committee of Ruijin Hospital, Shanghai Jiao Tong University School of Medicine, Shanghai, China” with written informed consent from all subjects.

**Table 1:** Demographic data on patients and normal controls.

	PD (*n* = 42)	NC (*n* = 40)	*p* value
Gender men/women	23M, 19F	21M, 19F	0.837
Age (years)	61.14 (7.13)	62.68 (5.73)	0.288
UPDRS-III (motor)	15.50 (6.36)	–	–
Disease duration (years)	2.96 (1.80)	–	–
Hoehn and Yahr (H-Y) score	1.33 (0.45)	–	–

*All data are given as mean (standard deviation). Chi-square test was used to test differences in gender distribution; two sample *t*-tests were used for other variables. PD = Parkinson’s disease; NC = Normal controls; UPDRS-III = Unified Parkinson’s Disease Rating Scale-Part III.*

### MRI Acquisition

Data acquisition was carried out on a GE 3.0 T HDxt MRI scanner with an 8-channel phased-array head coil. High-resolution T1-weighted MRI scans were acquired using a fast, spoiled gradient echo sequence (FSPGR) with the following parameters: repetition time (TR) = 5.528 ms, echo time (TE) = 1.74 ms, matrix size = 256 × 256, flip angle = 12°, slice thickness = 1 mm, 196 sagittal slices. A total of 210 volumes of resting-state fMRI data were acquired using an EPI sequence with the following parameters: TR = 2000 ms, TE = 30 ms, matrix size = 64 × 64, flip angle = 90°, voxel size = 3.75 × 3.75 × 4 mm^3^. Subjects were required to remain still and awake with eyes closed during the scan.

### Data Preprocessing

Preprocessing of fMRI data was performed using the DPARSF toolbox (Yan and Zang, 2010). The first 10 volumes of each functional time series were discarded considering instability of the initial MRI signal and adaptation of participants to the circumstance, leaving 200 volumes in total for processing as follows: slice-timing, realignment to the mean image, co-registration to the T1 image, motion correction, normalized to a standard template (Montreal Neurological Institute), reslicing to 3 × 3 × 3 mm^3^, spatial smoothing (FWMH = 6 mm) and band pass filtered (0.01–0.1 Hz). Finally, the nuisance covariates, including 24 head motion parameters, white matter signal, and CSF signal were regressed out. The resulting time courses were used for the following brain network construction and analysis. In order to limit the impact of head motion, we excluded the subjects with head motion greater than 2 mm of translation motion or more than 2 degrees of rotation.

### Definition of Functional Brain Networks

**FIGURE 1:**
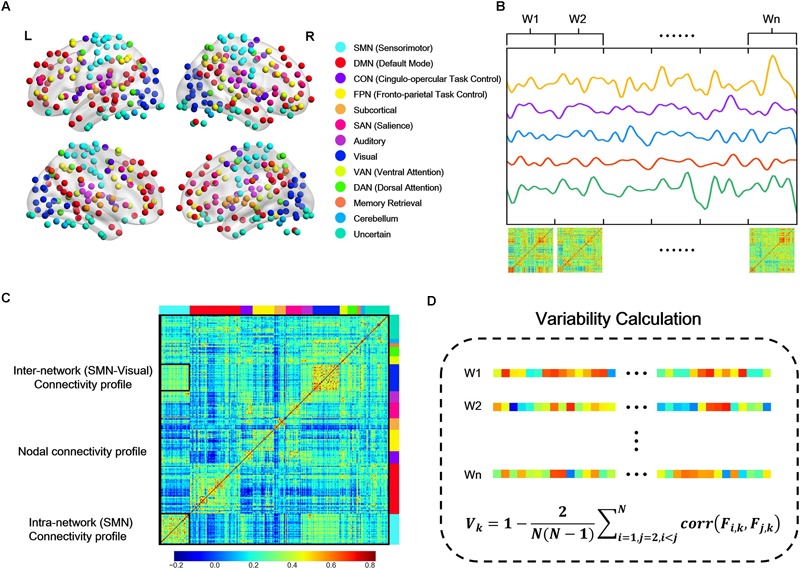
Schematic overview of the temporal variability calculation. **(A)** Network partition of 264 putative functional regions defined by Power et al. (2011) L, Left; R, right. **(B)** Each regional BOLD signal was segmented into non-overlapping windows, and the whole brain functional connectivity network was constructed using Pearson correlation of the representation BOLD time series in each window. **(C)** From the raw functional connectivity matrix in one time window, nodal connectivity profile is the vector that describes its connectivity with all the other nodes (e.g., the column marked by black line); intra-subnetwork connectivity profile is the upper triangle (due to symmetry) of the connectivity matrix within one subnetwork (e.g., SMN); inter-network connectivity profile is the connectivity matrix between two subnetworks (e.g., SMN-visual, as the block marked by a small black rectangular window). **(D)** Calculation of temporal variability. The nodal, intra- and inter-network connectivity profile in each window was unfolded into connectivity vectors, respectively, and the temporal variability was calculated as the average correlation coefficients of the connectivity vectors across different windows with a deduction from 1.

We adopted the spherical 264 functional Region of Interests (ROIs) defined by Power et al. (2011) across cortical, subcortical, and cerebellar structures, and extracted a representative BOLD time series by averaging signal in all voxels within each ROI. These brain regions were defined by resting-state FC mapping and multiple task fMRI meta-analysis, ensuring the functional significance of network nodes. In brain network analysis, each ROI was defined as a node, and the functional connectivity between ROIs was estimated from the corresponding representative BOLD time series. Consistent with previous study (Cole et al., 2013), the whole brain were mapped into 13 putative functional modules, including sensorimotor (SMN), default mode (DMN), cingulo-opercular (CON), fronto-parietal (FPN), subcortical, salience (SAN), auditory, visual, ventral attention (VAN), dorsal attention (DAN), memory retrieval, cerebellum, and uncertain networks (Figure 1A). This parcellation offers a comprehensive view for the study of functional brain modules, allowing to identify connectivity patterns and interactions between different modules. In this study, we would mainly report and discuss the results related to the 12 main networks but paying no attention to the “Uncertain” network, as it does not constitute specific cortical networks with explicable function.

For better interpretation for the anatomical location of specific regions, the Automated Anatomical Labeling (AAL) atlas (Tzouriomazoyer et al., 2002) was also used in this study.

### Temporal Variability

For each participant, dFC were computed based on sliding temporal window approach. Following the strategy in Zhang’s work (Zhang et al., 2016), we used segments of BOLD time series without overlapping to calculate temporal variability (nodal, intra- and inter-network variability). Briefly, the entire BOLD time series for each subject was divided into non-overlapping windows. Then within each window, connectivity between each pair of nodes was estimated by the Pearson’s correlation coefficients of the BOLD time series (Figure 1B).

#### Nodal Variability

To characterize the dynamic configuration of a specific region across all time windows, we calculated the temporal variability of each node (Mueller et al., 2013; Zhang et al., 2016). For a given region *k*, the connectivity profile at time window *i* can be denoted as *F_i,k_*, which is a vector with *M* values that describes the connection map based on the connectivity between region *k* with all other regions. Then the connectivity vectors in different windows were subsequently used to estimate the variability *V_k_* of brain region *k*, which is defined as one minus the average correlation of that region’s connectivity profile across all time windows (Mueller et al., 2013; Zhang et al., 2016), that is,

$$V_{k}=1-\frac{2}{N\left(N-1\right)}\sum_{i=1,j=2,i<j}^{N}corr\;(F_{i,k},F_{j,k}),$$

where *N* denotes the number of windows (Figure 1C,D). The second part of *V_k_* is the averaged correlation values between any two connectivity profiles derived from different time windows, indicating the similarity between connectivity profiles. A small value of variability *V_k_* indicates a high correlation of a node’s functional architecture across different time windows.

As for the parameters selection, previous studies suggested that window size around 30–60 s should be suitable to capture the resting-state dFC fluctuations and produce robust results (Keilholz et al., 2012; Li et al., 2014; Deng et al., 2016). And another study (Li et al., 2014) showed that changes of brain connectivity are not sensitive to the specific time-window length (in the range of 10–20 TRs, 20–40 s). We performed the variability analysis at different window length (*l* = 10, 11, 12, …, 20 volumes, corresponding to 20, 22, 24, …, 40 s, respectively), and found that variability obtained from different window lengths produced highly correlated results (*r* > 0.98, Supplementary Figure S1), indicating that this metric is not sensitive to the choice of window length. Therefore, in the following sections, we reported the results obtained with the window size of 20TRs (40 s).

#### Intra- and Inter-Network Variability

The connectivity patterns within and between subnetworks also fluctuate dynamically over a short period of time (Kiviniemi et al., 2011; Takamitsu et al., 2013; Ma et al., 2014). In order to assess the dynamic interactions within and between subnetworks and identify the system-level dynamic brain organization, we defined the intra- and inter- network variability in a way similar to the definition of nodal variability. For a subnetwork, all the intra-network connectivity in each window were unfolded into a long connectivity vector, and then the intra-network variability was estimated as the average correlation coefficients of the connectivity vectors across different windows with a deduction from 1 (Figure 1C,D). Intra-network variability characterizes the changes of connectivity within subnetwork over time. High value of intra-network variability means the functional architecture within the network is poorly correlated across different time windows, implying an unstable state. Similarly, for two subnetworks, all the connectivity between the two subnetworks were unfolded into a long connectivity vector, and then the inter-network variability was estimated as the average correlation coefficients of the connectivity vectors across windows with a deduction from 1 (Figure 1C,D). High value of inter-network variability means that the interactions between the two networks reconfigure frequently but not maintain a stable pattern.

### Statistical Analysis

Non-parametric permutation tests (Nichols and Holmes, 2002; Bassett et al., 2011) were applied to test the between-group differences in nodal, intra- and inter-network variability. In each permutation, each subject was randomly reassigned to one of a pair of groups with the same number of subjects as in the original patient and control groups. Then the nodal, intra- and inter-network variability were computed for each randomized group and the between-group difference for all the metrics was calculated, respectively. This randomization procedure was repeated 10000 times, resulting in a sampled null between-group difference permutation distributions for each metric. Finally, the *p*-value was then calculated as the proportion of total entries resulting from the permutation distribution that were greater than (or smaller than) the observed group effect.

Statistical analysis for head motion parameters and demographic measures were performed using two sample *t*-test. Spearman correlation analysis was performed between altered temporal variability and clinical variables of disease severity (UPDRS-III score and Hoehn and Yahr staging) at a threshold for statistical significance of *p* < 0.05. Statistical analysis of all the metrics were implemented using Matlab (version 2014a; MathWorks). Visualization of results were performed using Python.

### Validation Analysis

To evaluate the robustness of our main results, we conducted some validation analysis. (i) Parcellation scheme: given that the variability may be affected by the topological spatial constraints and definition of ROIs, we also constructed functional brain networks using an additional functional whole-brain parcellation scheme consisting of 268 ROIs (Shen et al., 2013) (referred to as Shen268 atlas), in which all the ROIs are assigned to eight different functional networks. (ii) Window length: to investigate the potential effects of window length, we performed the correlation analysis of temporal variability obtained at different window lengths, and also repeated the analysis with the average value of variability across different window lengths, following the strategy used in Zhang et al. (2016).

## Results

### Nodal Variability

We found significant nodal variability changes in PD patients across several regions. The nodal variability of precentral and postcentral gyrus (in SMN), middle occipital gyrus (in visual network), putamen (in subcortical network) and cerebellum in patients showed significantly increased variability (*p* < 0.005, 10000 permutations, uncorrected) compared to NCs (Table 2). While in this PD cohort, no nodes showed significantly decreased variability.

**Table 2:** Regions showing significant higher nodal variability in PD patients than normal controls.

ROI Index	MNI coordinate	Nodal Variability		*p*-value (uncorrected)	Subnetwork	Brain region (AAL atlas)
		NC	PD			
8	–37, –29, –26	0.8033	0.8508	0.0033	Uncertain	Fusiform_L
28	20, –29, 60	0.7350	0.8013	0.0005	SMN	NA
34	–21, –31, 61	0.7486	0.8042	0.0035	SMN	Postcentral_L
41	38, –17, 45	0.7229	0.7980	0.0008	SMN	Precentral_R
147	–28, –79, 19	0.7360	0.7817	0.0044	Visual	Occipital_Mid_L
227	–22, 7, –5	0.8031	0.8474	0.0026	Subcortical	Putamen_L
232	–31, –11, 0	0.8072	0.8565	0.0029	Subcortical	Putamen_L
245	22, –58, –23	0.7492	0.8034	0.0046	Cerebellum	Cerebellum

### Intra-Network Variability

**FIGURE 2:**
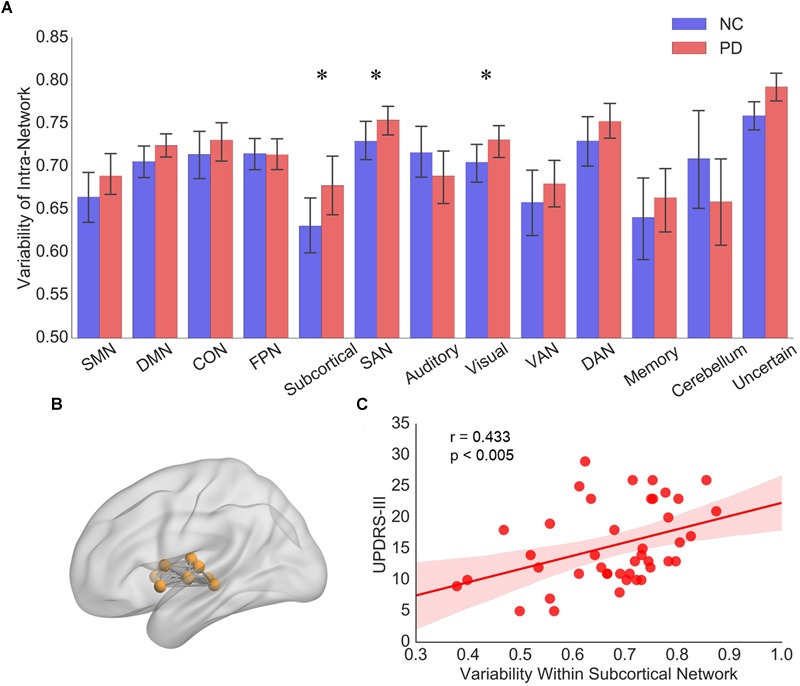
**(A)** The intra-network variability of 13 subnetworks for PD patients and NCs, respectively. Error bars represent mean and standard errors of the two groups, respectively. ^∗^*p* < 0.05. **(B)** Topographic representation of the nodes and connectivity within subcortical network. **(C)** Scatter plots of intra-network variability of subcortical network with respect to the UPDRS-III score in PD patients. Each dot indicates one subject. Linear regression line with 95% confidence interval for best-fit line (shading area), as well as *r* and *p* values (Spearman’s correlation coefficient) are provided.

Among the 13 subnetworks, we found that subnetworks including subcortical network, SAN and visual network tended to display greater intra-network variability in PD than NCs (*p* < 0.05, 10000 permutations, Figure 2A). Only the variability of intra-subcortical network showed a positive correlation with UPDRS-III by spearman correlation analysis (Figure 2B,C).

### Inter-Network Variability

#### Distributed Difference of Inter-Network Variability

**FIGURE 3:**
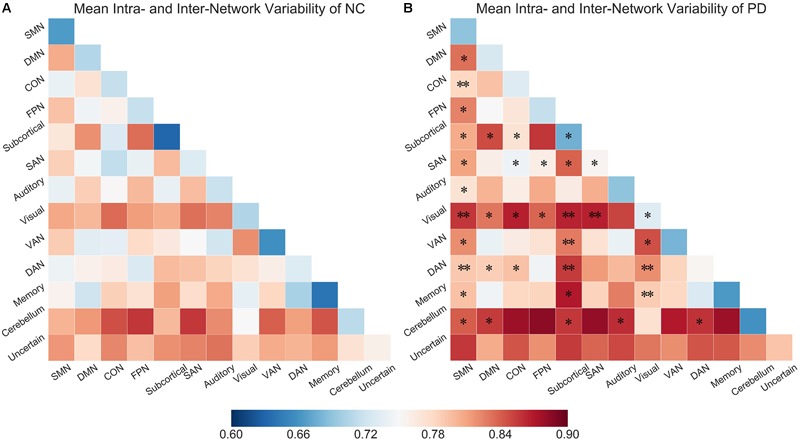
**(A)** Mean intra- and inter-network variability matrix of NCs. **(B)** Mean intra- and inter- network variability matrix of PD patients. Asterisks (^∗^) labeled on the PD matrix indicate the subnetwork pairs showing significantly increased intra- or inter-network variability in PD patients compared than control subjects. ^∗^*p* < 0.05, ^∗∗^*p* < 0.005.

We explored inter-network variability among all subnetwork pairs. Figure 3A,B show the mean intra- and inter- network variability matrices in NCs and PD patients, respectively. Generally, the intra-network variability of a particular subnetwork demonstrated a relatively lower value than the inter-network variability of that subnetwork with other subnetworks, respectively in both PD and NC groups (Figure 3, NC group: all *p*-values no larger than 0.0181, 10000 permutations; PD group: all *p*-values no larger than 0.0001, 10000 permutations). Between group comparisons for the variability matrices revealed significantly greater inter-network variability in PD patients compared with NCs in several subnetwork pairs associated with SMN, visual, subcortical networks and cerebellum.

#### Correlation Between Inter-Network Variability and UPDRS-III

We further identified the subnetwork pairs which showed significant correlation (*p* < 0.05, Spearman correlation) to the clinical status. To ensure correlations are not driven by a few extreme values, we removed outliers in the data prior to performing linear regression. We defined an outlier as a value outside of 3 standard deviation from the group mean. Performing regression with and without outliers actually did not qualitatively change the results.

**FIGURE 4:**
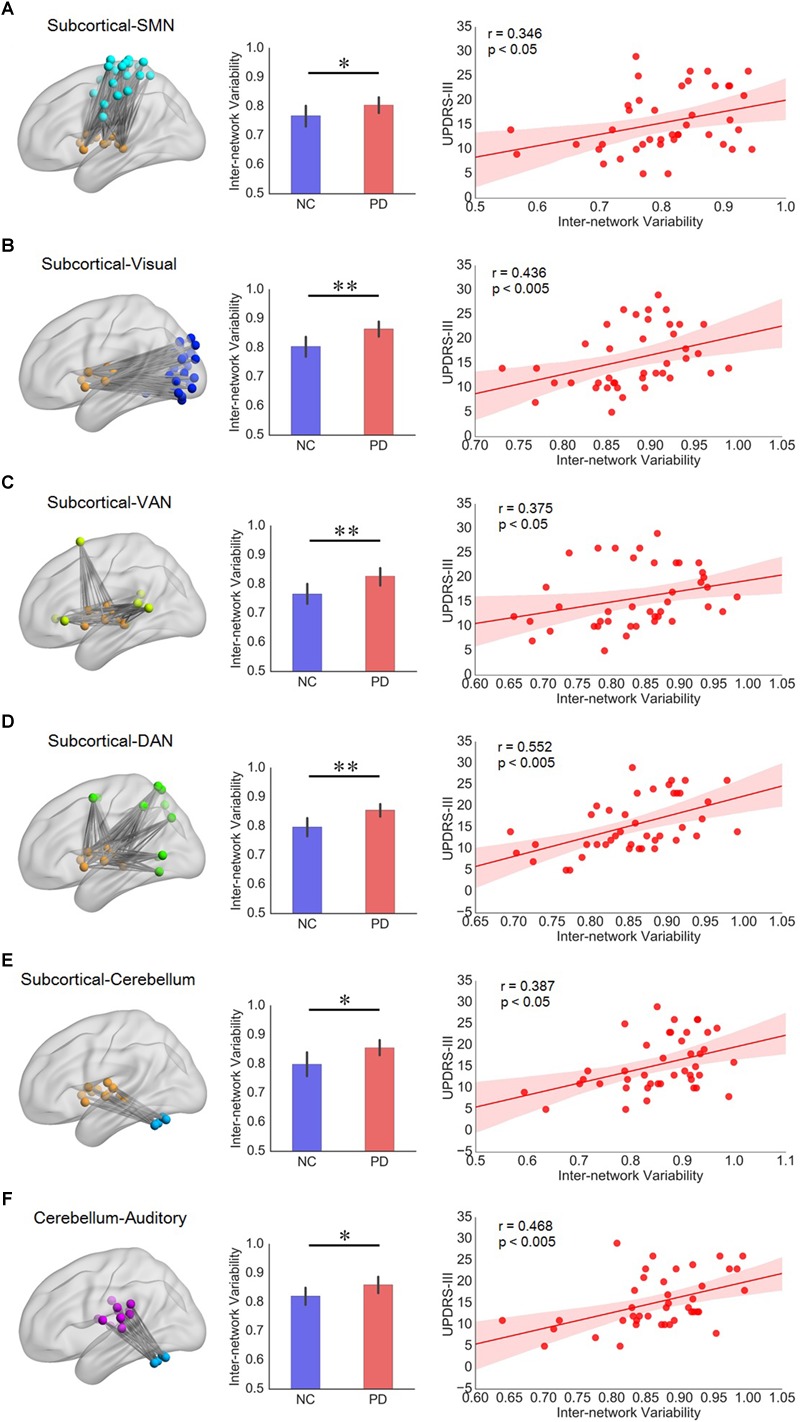
**(A–E)** Subnetwork pairs associated with subcortical network had increased inter-network variability as well as significant correlation with UPDRS-III. **(F)** The cerebellum-auditory subnetwork pair which had increased inter-network variability as well as significant correlation with UPDRS-III in PD patients. For each row from A to F, the left panel is the topographic representation of the nodes and the inter-network connectivity within the corresponding subnetwork pairs, respectively; the middle panel is the boxplot of inter-network variability for NC and PD groups, respectively; and the right panel is the scatterplot of the inter-network variability with respect to the UPDRS-III score for PD patients, with each dot indicating one subject, excluding outliers (outside of 3 standard deviation from the group mean). Error bars represent mean and standard errors of the two groups, respectively. Linear regression line with 95% confidence interval for best-fit line (shading area), as well as *r* and *p* values (Spearman’s correlation coefficient) are provided. ^∗^*p* < 0.05, ^∗∗^*p* < 0.005.

Significant correlation between inter-network variability and UPDRS-III mainly appeared in subnetwork pairs associated with subcortical network and cerebellum (Supplementary Figure S5). Compared with NCs, PD group had greater inter-network variabilities for the subnetwork pairs of subcortical network with respect to sensorimotor, visual, ventral attention, dorsal attention, and cerebellum networks, and their variability also had significant positive correlation with UPDRS-III in PD patients (Figure 4A–E). In addition, the inter-network variability for subnetwork pairs of cerebellum and auditory network also showed group difference as well as significant positive correlation with UPDRS-III (Figure 4F). We have also evaluated the correlation between temporal variability and H-Y scores, while the results showed that the nodal/intra- and inter-network variability had a weak correlation (all *r* < 0.23, all *p* > 0.14) with H-Y scores.

### Validation Results

We validated our main findings using different analysis strategies, involving sliding window lengths (Supplementary Table S1 and Supplementary Figures S1–S4), parcellation schemes (Supplementary Table S2 and Supplementary Figures S6–S8). We found that the temporal variability including nodal, intra- and inter- network variability estimated from windows of different lengths were highly correlated, indicating that these metrics is not sensitive to the choice of window length. For further validation of the results, we also used the average variability across different window lengths for the same analysis procedures, the main results are highly similar with those at window length of 20TRs. For the results obtained at Shen268 atlas, we observed similar pattern of variability difference of two groups, which were mainly located at Subcortical-cerebellum, Motor, and Visual (Visual I, Visual II, Visual association) networks. Besides, the significant correlation between temporal variability and UPDRS-III also mainly appeared in subnetwork pairs associated with Subcortical-cerebellum network. These results replicated the main findings obtained with Power-264 atlas.

## Discussion

In the present study, we investigated the variability of the dynamic functional brain network of PD patients at nodal, intra- and inter-network levels. Our results demonstrated that PD patients exhibited increased nodal variability involving precentral and postcentral areas, occipital area, putamen, and cerebellum. Moreover, PD patients demonstrated significantly increased intra-network variability within subcortical, salience and visual networks, as well as distributed increase in inter-network variability of several subnetwork pairs. Furthermore, the intra-network variability of subcortical network and the inter-network variability of subcortical network with respect to SMN, visual network, VAN, DAN and cerebellum showed significant correlation with the clinical score UPDRS-III, suggesting the special role of subcortical network in functional abnormality of PD. Besides, the inter-network variability of the cerebellum-auditory pair also had significant correlation with the clinical score UPDRS-III. These results suggest that PD patients showed widespread functional network abnormalities in term of temporal variability, and the abnormal temporal variability also correlated with clinical manifestations and thus offering new insights in understanding PD.

### Distributed Variability Changes in PD Patients

At the nodal and intra-network level, we found a widespread pattern of increased variability in PD patients, encompassing sensorimotor (SMN), visual, subcortical networks and cerebellum. A similar pattern was also observed in ROIs defined by the Shen268 atlas, that is, the variability difference was mainly located in Subcortical-cerebellum, Motor, and Visual (Visual I, Visual II, Visual association) subnetworks (Supplementary Table S2 and Supplementary Figures S6,S7). Previous study has reported that the primary sensorimotor area in normal subjects changed little over time (Power et al., 2011; Bassett et al., 2013). Conversely, in this study, the precentral and postcentral areas (in sensorimotor network) revealed higher variability in patients, unveiling that nodes in SMN of PD patients demonstrated a non-stationary functional configuration with other regions. In fact, several studies have identified abnormal functional connectivity in SMN which is indicative of impaired sensorimotor integration occurred in PD (Lewis and Byblow, 2002; Tessitore et al., 2014). Besides, the increased variability has also been observed in visual network both at nodal and intra-network levels. Indeed, visual network is another major complex sensory domain affected by PD, and PD may lead to visual hallucinations, perceptual, executive and sleep dysfunction (Archibald et al., 2011; Wu et al., 2011; Weil et al., 2016). These results together suggest that the altered dFC patterns observed in our study may be related to the deficits in these sensory regions. Moreover, the salience network, mainly comprised of the anterior insula, is a central hub involved in integrating the somatosensory and cognitive-affective information to guide behavior (Kurth et al., 2010). The increased intra-salience network variability found in our data corroborated previous findings of reduced hub role (Tinaz et al., 2016) and abnormal activation patterns in insula in PD patients (Christopher et al., 2014, 2015). In addition, there were other between-group differences in regional or inter-subnetwork variability associated with subcortical network and cerebellum, many of which have been suggested to be related to structural or functional impairment in PD patients (Brooks et al., 2010). Taken together, the abnormality of regional variability identified in our study indicates altered dFC across distributed nodes and subnetworks, reflecting those regions are at an unstable state.

For the inter-network variability, we found that the inter-motor variability was higher than intra-motor variability, which is consistent with previous studies (Elton and Gao, 2015) that lower variability within subnetwork may maintain a more stable configuration. In addition, significant higher inter-network variability was found across distributed subsystem pairs. The varying interactions between subsystems may be tied to functional coordination between subnetworks (Zalesky et al., 2014), the increased inter-network variability in PD patients implies an unstable state and more dynamic interactions. Our observations was consistent with Kim et al. (2017), which reported an increase in the number of transitions between states and higher variability in global efficiency of brain network of PD patients, implying a less efficient and more unstable information transfer within/between functional subnetworks. Furthermore, substantial efforts have been made in previous studies to elucidate the neural basis of PD, with mounting evidence indicating deficits of distributed brain networks and wide-spread white matter damage in PD (Brooks and Pavese, 2011; Canu et al., 2016; Koshimori et al., 2016). Our results putatively reflect the abnormal global integration of the brain networks in PD from the dynamic perspective.

### Variability Associated With Subcortical Network

Despite the distributed alteration of temporal variability in PD patients, the variability showing significant correlation with clinical score was particularly associated subcortical network (Supplementary Figure S5), suggesting its clinical significance in PD. Results obtained with the Shen268 atlas showed a similar trend, which were mainly associated with the Subcortical-cerebellum subnetwork (Supplementary Figure S8). At nodal and intra-network levels, a higher nodal variability in putamen and intra-network variability within subcortical network was observed, which is compatible with previous study highlighting the severely affected striatal dopamine depletion in putamen and reduced connectivity between striatal and thalamus within subcortical network (Hacker et al., 2012). At the inter-network level, the increased inter-network variability between subcortical network and cortical networks (sensorimotor, visual, and attention network) in PD patients also correlated with worsening motor symptoms in PD. All these cortical networks are known to play a critical role in the pathogenesis of PD (Kim et al., 2017), and the dysfunction of cortico-BG-thalamo-cortical circuit associated with motor deficits is well documented in PD patients (Alexander et al., 1986). More specifically, altered functional connection and integration of subcortical to sensorimotor and visual networks have been reported in PD (Tremblay et al., 2010; Hacker et al., 2012; Wymbs et al., 2012). Connectivity changes and dysfunctional integration in attention work (including dorsal attention network (DAN) and ventral attention network (VAN)) have also been reported in PD patients with mild cognitive impairment and visual hallucinations, respectively (Shine et al., 2013a; Baggio et al., 2015). Here the higher level variability of FC profile in subcortical-cortical coupling may therefore provide further information for the prediction of disease severity. Furthermore, a significantly increased variability of cerebellum-subcortical coupling in PD patients was positive associated with UPDRS-III in our study. According to Bostan et al. (2013), the reciprocal connections between basal ganglia and the cerebellum provide the anatomical substrate for the cerebellar contributions to a wide range of behaviors. The increased variability pattern between cerebellum and subcortical network, could also support the markedly lower striatal connectivity with cerebellum in PD group reported in Hacker et al. (2012), thus reinforcing the relevance to parkinsonism of the described circuits connecting the cerebellum to the basal ganglia (Hoshi et al., 2005; Bostan et al., 2010).

### Variability Associated With Cerebellum

Interestingly, in the present study, the node in the cerebellum showed significantly increased nodal variability in PD patients. Studies have shown that the cerebellum is involved in motor coordination (Thach, 1998) as well as integration of motor and cognitive networks. Pathological changes in the cerebellum have been reported in a previous study (Wu and Hallett, 2013). Considering this, our results revealed an abnormal dynamic connectivity profile between nodes in the cerebellum with other regions, which may be related to the dysfunction of cerebellum in PD patients.

We also found that a significantly increased inter-network variability between cerebellum and cortical network (auditory network) showed positive correlation with UPDRS-III. This emphasizes that the unstable dynamic interaction between the cerebellum and auditory network may also relate to the motor symptom of PD. Similar to basal ganglia in subcortical network, cerebellum has also shown anatomical loops between cerebellum and nearly all cortical subnetworks, suggesting its high global brain connectivity (Middleton and Strick, 1994; Kelly and Strick, 2003; Cole et al., 2010). Combined with the observations that the inter-network variability between subcortical network and other cortical subnetworks as well as cerebellum were also correlated with clinical score (UPDRS-III) noted above, our results uncover the fact that cerebellum also serve as an important role in PD pathology.

In fact, the subcortical network in this study (mainly comprised of basal ganglia and thalamus) and the cerebellum are densely interconnected at the subcortical level and formed an integrated network (Bostan and Strick, 2018). These subcortical systems support the convergence of diverse cortical and subcortical afferents, as well as neuromodulatory signals from the brainstem, thus serving as a hub for large-scale network integration in the human brain (Bell and Shine, 2016). Previous studies revealed that pathological lesions of most disorders were concentrated in hub regions, especially in the striatum and thalamus, implying that subcortical hubs represent key pathological foci across multiple brain disorders (Crossley et al., 2014). Evidence from previous studies has shown that cortico-subcortical circuits are linked to a diverse range of limbic, cognitive and motor control functions (Chudasama and Robbins, 2006; Pennartz et al., 2009). PD is a neurodegenerative disorder characterized by severe and early subcortical pathology as well as clinical impairments extend across cognitive and motor domains. Our results provide further evidence of a subcortical hub by capturing the dynamic variability changes in the subcortical network of PD, suggesting that the subcortical dysfunction may contribute to pathological changes in PD.

### Limitations

Several limitations of this study should be recognized. First, motor manifestations of PD subjects are evaluated only using the motor portion of UPDRS without other neuropsychological tests for the diagnosis of PD. There are other substantial non-motor symptoms such as cognitive impairment, autonomic dysfunction and sleep disorders experienced by PD patients (Wu et al., 2011). Future work could investigate variability metrics and their association with other neuropsychological scores and cognitive performance to establish the relationship between dynamic analysis and clinical diagnosis. Second, PD is a heterogeneous disorder with different symptoms and functional connectivity patterns (Zhang et al., 2015). Due to the diversity of possible status of drug use, personality, or genetic factors in our data and a large number of comparisons, some results didn’t pass a false discovery rate (FDR) for multiple comparisons correction. Accordingly, we reported the results by setting a relative low significance level (*p* < 0.005) in nodal variability and different statistic levels (*p* < 0.05 and *p* < 0.005) in intra- and inter-network variability. Actually, a more heterogeneous status in the patients’ group will most likely lead to higher statistical variance, rendering it more difficult to detect significant functional connectivity differences between patients and controls. Therefore, a larger sample size and more strict inclusion criteria will be needed in further study to reveal temporal variability differences.

## Conclusion

This study reported alterations of temporal variability in PD patients at nodal, intra- and inter-network levels. Our analysis showed that PD patients have higher nodal variability in precentral and postcentral area, middle occipital gyrus, putamen, cerebellum as well as the increased intra- and inter-motor variability across several distributed subnetworks compared with normal controls. Moreover, the higher intra- and inter-network variability associated with the subcortical network and cerebellum showed significant correlation with UPDRS-III motor score, suggesting its clinical significance. Our results suggest that temporal variability of functional connectivity profile can detect the aberrant dynamic connectivity patterns, which were associated with the clinical deficits in PD and thus may deepen our understanding of the disease.

## Data Availability Statement

The dynamic functional connectivity matrix used in our analysis are available from the authors upon reasonable request.

## Author Contributions

HZ, JH, JS, and HL conceived and designed the study. HZ, LD, LC, and PS analyzed the data. JH, FY, NH, and HL performed the data acquisition. JS and HL supervised the study. HZ drafted the manuscript. JS, ST, and NH involved manuscript review and critique. All authors approved the final manuscript.

## Conflict of Interest Statement

The authors declare that the research was conducted in the absence of any commercial or financial relationships that could be construed as a potential conflict of interest.
