# Supplementary material for: Abnormal Dynamic Functional Connectivity Associated With Subcortical Networks in Parkinson’s Disease: A Temporal Variability Perspective
Source: Front Neurosci. 2019 Feb 19;13:80. doi: 10.3389/fnins.2019.00080 (PMC6389716; doi:10.3389/fnins.2019.00080)
Supplement: Supplementary file 1 [file Table_1.docx]

Supplementary Material

**Choice of window length**

To validate the effects of window length on our main results, two strategies were adopted. First, we evaluated the correlation of variability obtained at different window lengths (10TRs, 11TRs, 12TRs, …, 20TRs, corresponding to 20, 22, 24, …, 40 s), and found the values were highly correlated (all the r > 0.98 for NC group and PD group). We take the length of 10TRs, 15TRs, 20TRs, corresponding to 20s, 30s, 40s as examples to show the correlation of temporal variability (nodal variability, intra- and inter- network variability) for normal controls and PD patients (**Supplementary Figure 1**).


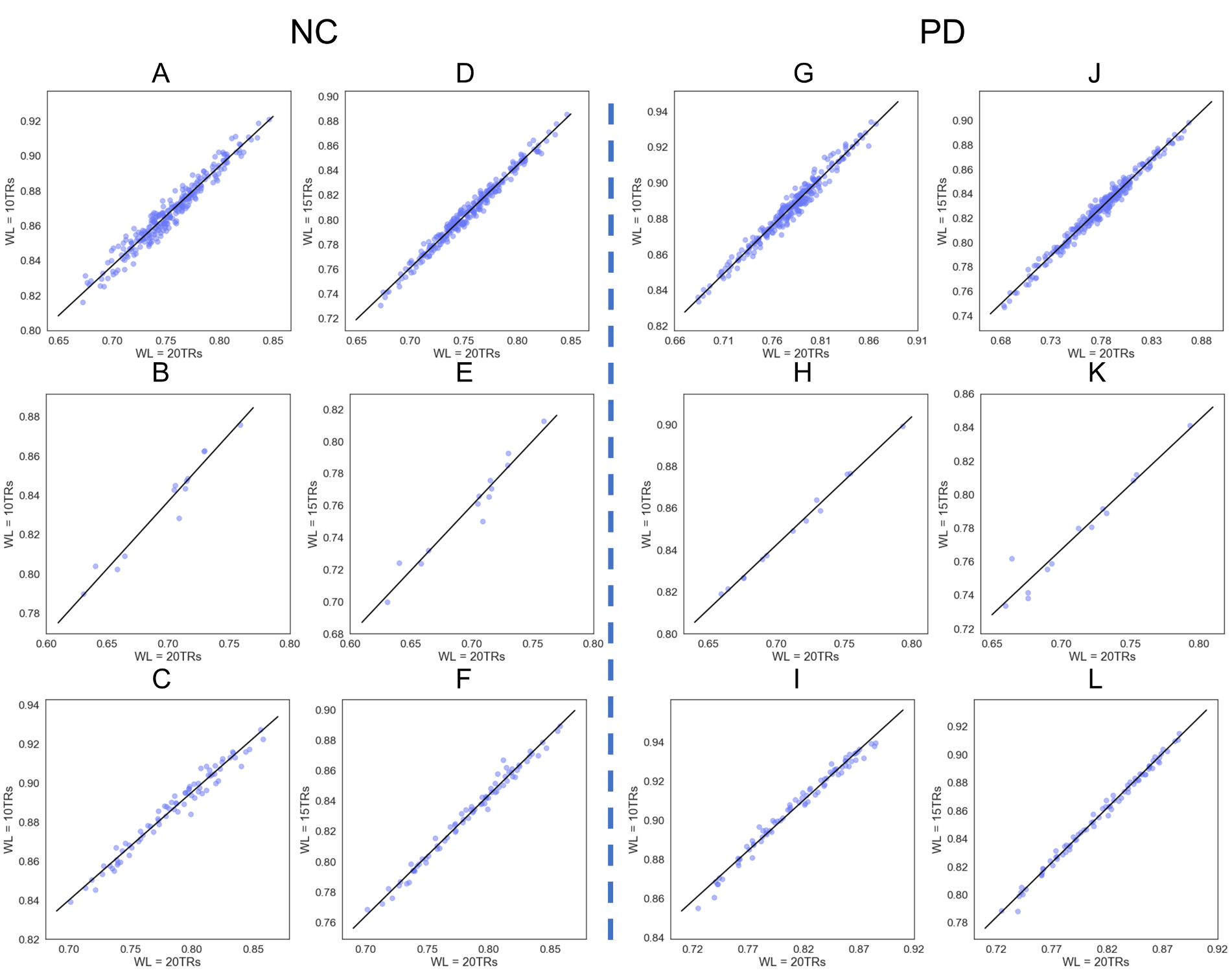


**Supplementary Figure 1.** Scatter plots of temporal variability obtained at window length of 10TRs (20s) and 15TRs (30s), and 20TRs (40s) for normal controls (A-F, left) and PD patients (G-L, right) respectively. From top to bottom line, the scatter plots are the results of nodal variability, intra-network variability, and inter-network variability respectively. Each dot in the figure represents a node, or a subnetwork, or a subnetwork pair respectively, and the variability is averaged over control/patient group. In the scatter plot, variability obtained at different window length shows high correlation (all r > 0.98), suggesting that temporal variability defined in the paper are not sensitive to the choice of window length.

Second, For consistency and comparability with previous work ([Zhang et al., 2016](#_ENREF_2)), we also calculated the average value of variability across different window lengths (10TRs, 11TRs, 12TRs, …, 20TRs, corresponding to 20, 22, 24, …, 40 s). The nodes with significant difference in the average value of variability across different window lengths were summarized in **Supplementary Table 1**.

**Supplementary Table 1.** Regions showing significant higher nodal variability in PD patients than normal controls. Here the nodal variability value denotes the average of nodal variability across different window lengths.

| ROI Index | MNI coordinate | Nodal Variability | | p-value (uncorrected) | Subnetwork | Brain region  (AAL atlas) |
| --- | --- | --- | --- | --- | --- | --- |
|  |  | NC | PD |  |  |  |
| 8 | -37,-29,-26 | 0.8485 | 0.8875 | 0.0018 | Uncertain | Fusiform_L |
| 28 | 20,-29,60 | 0.7929 | 0.8475 | 0.0008 | SMN | NA |
| 41 | 38,-17,45 | 0.7860 | 0.8416 | 0.0028 | SMN | Precentral_R |
| 141 | 17,-91,-14 | 0.8228 | 0.8644 | 0.0046 | Uncertain | Lingual_R |
| 171 | -28,-79,19 | 0.7951 | 0.8400 | 0.0043 | Visual | Occipital_Mid_L |
| 192 | 44,-53,47 | 0.7689 | 0.8146 | 0.0025 | FPN | Parietal_Inf_R |
| 211 | 34,16,-8 | 0.7723 | 0.8201 | 0.0035 | Salience | Insula_R |
| 231 | 29,1,4 | 0.8175 | 0.8566 | 0.0048 | Subcortical | Putamen_R |
| 232 | -31,-11,0 | 0.8510 | 0.8909 | 0.0029 | Subcortical | Putamen_L |
| 245 | 22,-58,-23 | 0.7996 | 0.8489 | 0.0013 | Cerebellum | Cerebellum |


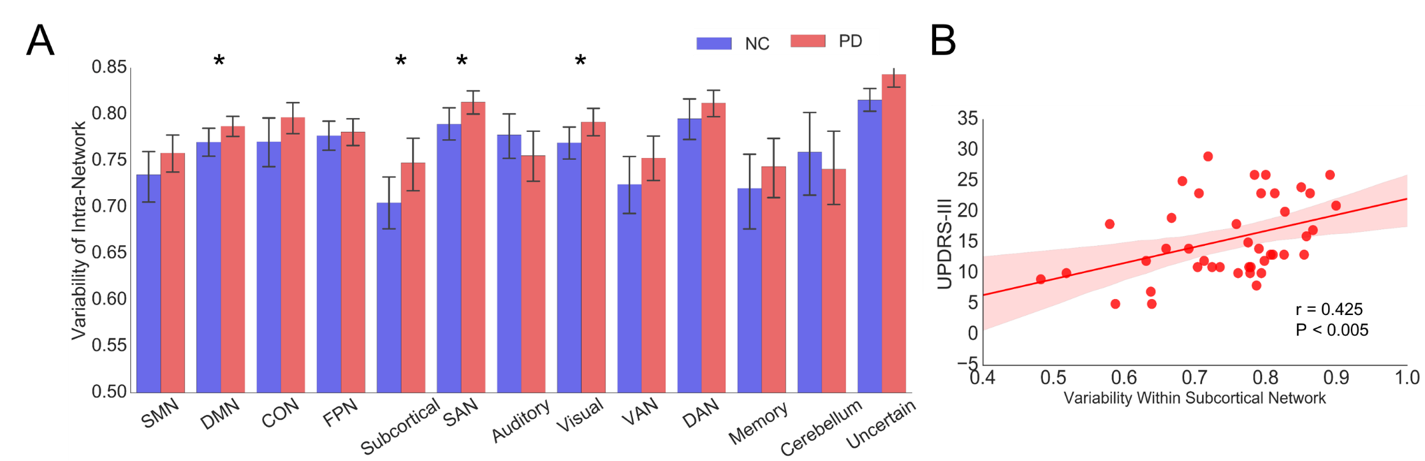


**Supplementary Figure 2.** (A) The intra-network variability of 13 subnetworks for PD patients and NCs respectively. Error bars represent mean and standard errors of the two groups respectively. *p<0.05, **p<0.005. (B) Scatter plots of intra-network variability of subcortical network with respect to the UPDRS-III score in PD patients. Each dot indicates one subject. Linear regression line with 95% confidence interval for best-fit line (shading area), as well as *r* and *p* values (Spearman’s correlation coefficient) are provided. For each subnetwork, the intra-network variability was averaged by the value acquired across different window lengths (10TRs, 11TRs, 12TRs, …, 20TRs, corresponding to 20, 22, 24, …, 40 s).


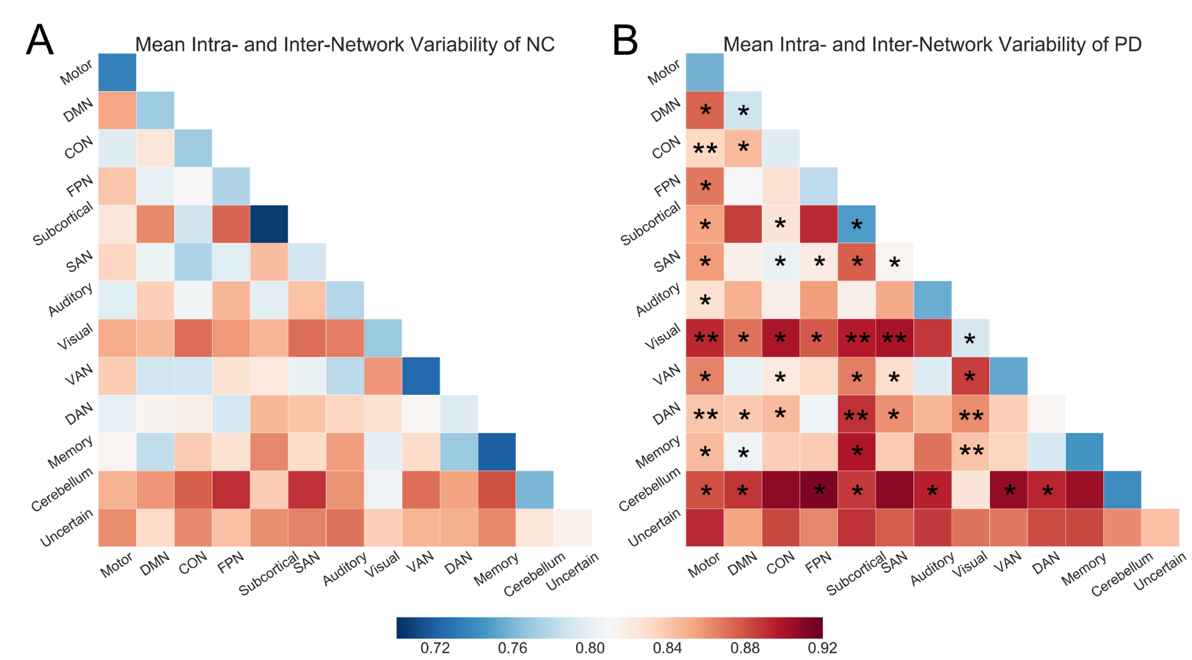


**Supplementary Figure 3.** Intra- and inter-network variability acquired at average value of different window lengths. (A) Mean intra- and inter-network variability matrix of NCs. (B) Mean intra- and inter- network variability matrix of PD patients. Asterisks (*) labeled on the PD matrix indicate the subnetwork pairs showing significantly increased intra- or inter-network variability in PD patients compared than control subjects. *p<0.05, **p<0.005

**
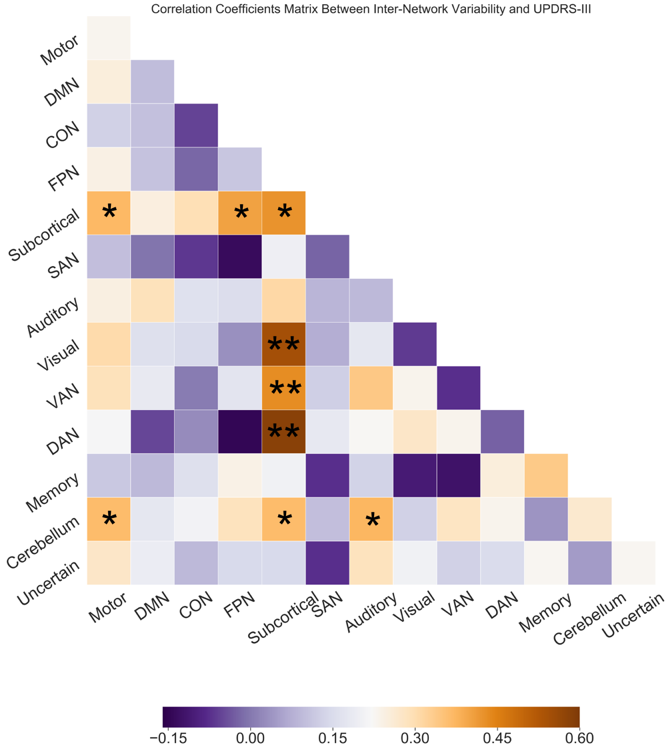
**

**Supplementary Figure 4.** Correlation coefficients matrix between average value of inter-network variability across different window lengths and UPDRS-III. Asterisks (*) labeled on the matrix indicate the subnetwork pairs whose average inter-network variability across different window lengths showed significant correlation (p<0.05, **p<0.005, Spearman correlation) with UPDRS-III in PD patients.

**
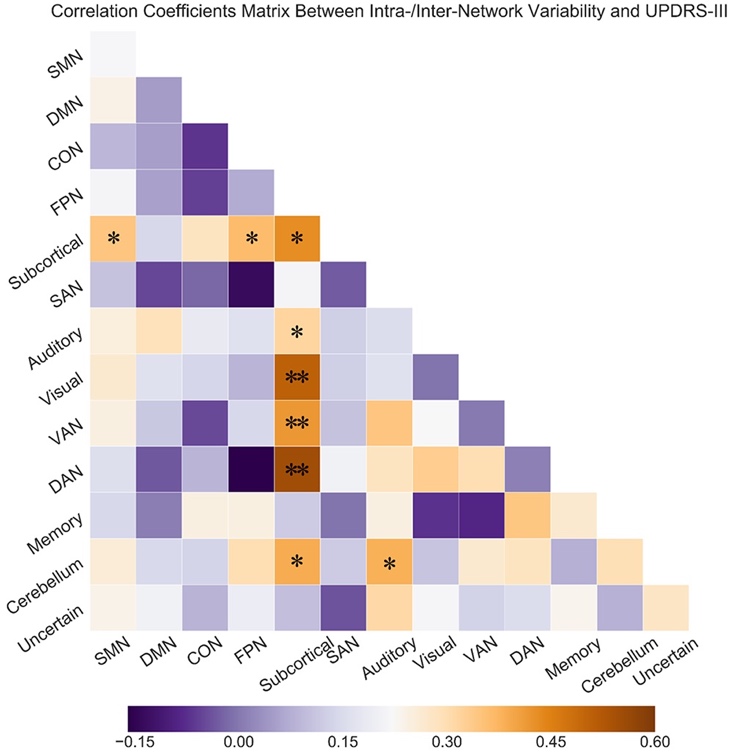
**

**Supplementary Figure 5.** Correlation coefficients matrix between inter-network variability and UPDRS-III (WL = 20TRs (40s)). Asterisks (*) labeled on the matrix indicate the inter-network variability of subnetwork pairs showing significant correlation (p<0.05, **p<0.005, Spearman correlation) with UPDRS-III in PD patients.

**Parcellation scheme**

We use another functional brain atlas defined by Shen et al. (Shen268 atlas) ([Shen et al., 2013](#_ENREF_1)) to perform the data analysis. This atlas consists of 268 nodes covering the whole brain and the nodes were further grouped into eight functional subnetworks (Medial frontal, Frontoparietal, Default mode, Subcortical-cerebellum, Motor, Visual I, Visual II and Visual association). In consistent with our main text, we showed the results obtained with the window size of 20TRs (40s) (**Supplementary Table 2**).

**Supplementary Table 2.** ROIs showing significant higher nodal variability in PD patients than normal controls. ROIs are defined by Shen268 atlas. For simplicity, here we reported the nodes with significant level at p < 0.001 before FDR correction.

| ROI Index | p-value | Subnetwork | Nodal Variability | |
| --- | --- | --- | --- | --- |
|  |  |  | NC | PD |
| 26 | 6.00E-04 | Motor | 0.680839506 | 0.75078958 |
| 42 | 5.00E-04 | Visual I | 0.717930865 | 0.785682522 |
| 65 | 0.00079992 | Medical Frontal | 0.713628861 | 0.770586059 |
| 110 | 0.00079992 | Subcortical-cerebellum | 0.677699691 | 0.755226885 |
| 119 | 0.00039996 | Subcortical-cerebellum | 0.689117507 | 0.784355711 |
| 172 | 0.00019998 | Motor | 0.685668721 | 0.762259842 |
| 178 | 0.00069993 | Subcortical-cerebellum | 0.707156577 | 0.763046722 |
| 209 | 0.00089991 | Visual Association | 0.705097067 | 0.766265477 |
| 218 | 1.00E-03 | Motor | 0.696205098 | 0.768029207 |
| 233 | 0.0009999 | Subcortical-cerebellum | 0.733303296 | 0.796593537 |
| 254 | 0.00069993 | Subcortical-cerebellum | 0.677328494 | 0.759746483 |


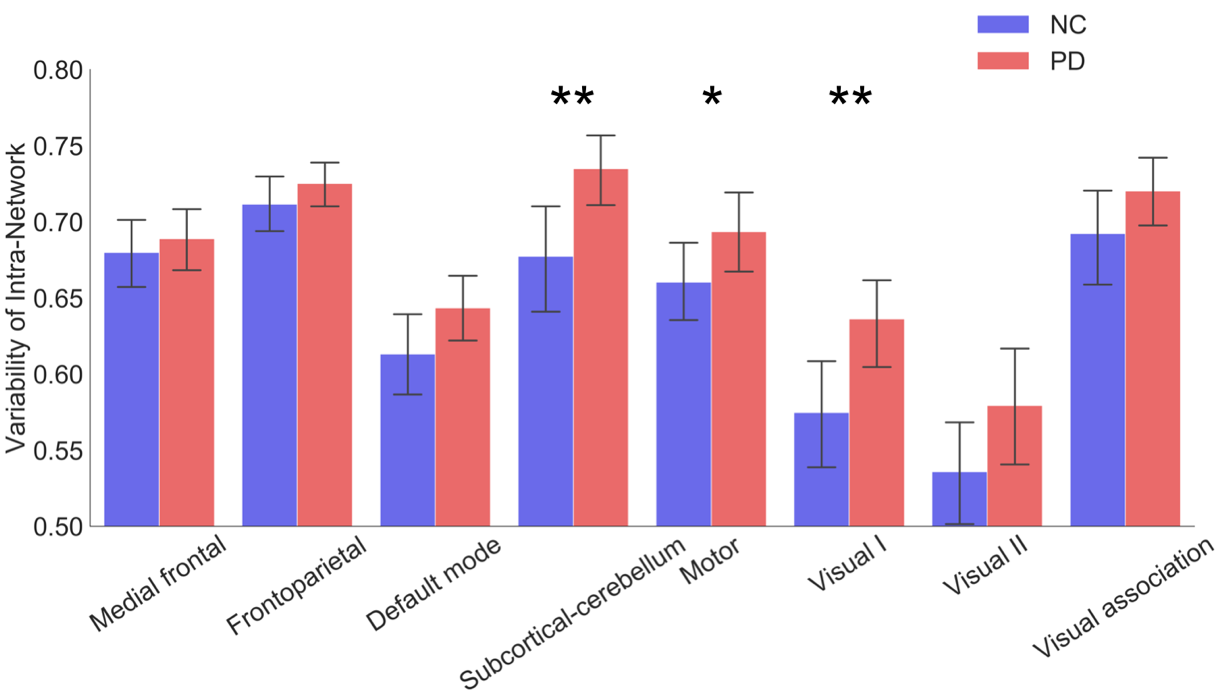


**Supplementary Figure 6.** The intra-network variability of eight subnetworks for PD patients and NCs derived from Shen268 atlas. Error bars represent mean and standard errors of the two groups respectively. *p<0.05, **p<0.005.


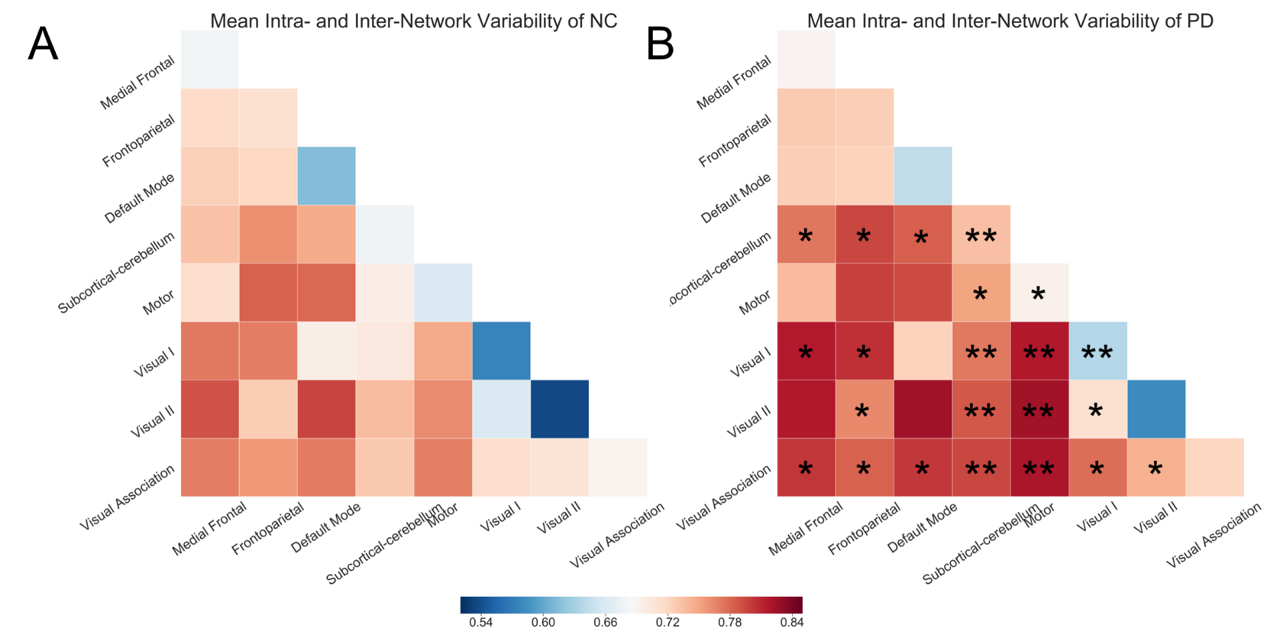


**Supplementary Figure 7.** Intra- and inter-network variability derived from Shen268 atlas. (A) Mean intra- and inter-network variability matrix of NCs. (B) Mean intra- and inter- network variability matrix of PD patients. Asterisks (*) labeled on the PD matrix indicate the subnetwork pairs that showed significantly increased intra- or inter-network variability in PD patients compared than control subjects. *p<0.05, **p<0.005


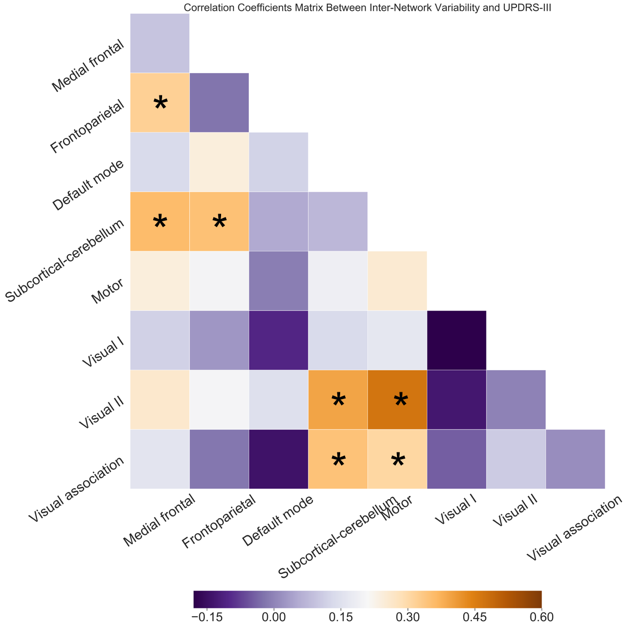


**Supplementary Figure 8.** Correlation coefficients matrix between inter-network variability and UPDRS-III derived from Shen268 atlas. Asterisks (*) labeled on the matrix indicate the inter-network variability of subnetwork pairs that showed significant correlation (p<0.05, Spearman correlation) with UPDRS-III in PD patients.

Shen, X., Tokoglu, F., Papademetris, X., and Constable, R.T. (2013). Groupwise whole-brain parcellation from resting-state fMRI data for network node identification. *Neuroimage* 82(2)**,** 403-415. doi: 10.1016/j.neuroimage.2013.05.081.

Zhang, J., Cheng, W., Liu, Z., Zhang, K., Xu, L., Ye, Y., et al. (2016). Neural, electrophysiological and anatomical basis of brain-network variability and its characteristic changes in mental disorders. *Brain* 139(8)**,** 2307-2321. doi: 10.1093/brain/aww143.
